# Validation of the Proposed Definition for Complicated Coagulase-negative Staphylococcal Bacteremia

**DOI:** 10.1093/cid/ciaf119

**Published:** 2025-03-13

**Authors:** Matthaios Papadimitriou-Olivgeris, Laurence Senn, Benoit Guery

**Affiliations:** Infectious Diseases Service, Lausanne University Hospital and University of Lausanne, Lausanne, Switzerland; Infectious Diseases Service, Institut Central des Hôpitaux and Hospital of Valais, Sion, Switzerland; Infectious Diseases Service, Lausanne University Hospital and University of Lausanne, Lausanne, Switzerland; Infection Prevention and Control Unit, Lausanne University Hospital and University of Lausanne, Lausanne, Switzerland; Infectious Diseases Service, Lausanne University Hospital and University of Lausanne, Lausanne, Switzerland

**Keywords:** coagulase negative staphylococci, source control, infectious diseases consultation, *Staphylococcus epidermidis*, complicated bacteremia

## Abstract

**Background:**

A new definition for complicated coagulase-negative staphylococcal (CoNS) bacteremia was recently proposed. The aim of this study was to identify predictors of mortality in patients with CoNS bacteremia and evaluate the proposed definition of complicated bacteremia.

**Methods:**

This retrospective study was conducted at the Lausanne University Hospital, Switzerland (2015–2023) and included adult patients with CoNS bacteremia.

**Results:**

During the study period, 326 episodes of CoNS bacteremia were included, with 250 (77%) episodes involving *Staphylococcus epidermidis*. Most infections were catheter-related bacteremias (233 episodes; 68%). Based on the proposed definition, 195 (60%) episodes had complicated disease. The overall 30-day mortality was 9% (29 episodes). Infectious diseases (ID) consultation was provided within 48 hours from bacteremia onset in 285/326 (87%) episodes. Source control was deemed necessary in 275 (84%) episodes and was performed within 48 hours in 167/275 (61%) episodes. No difference on 30-day mortality was observed among complicated and uncomplicated disease (10% vs 7%; *P* = .327). The Cox multivariable regression model showed that a Charlson comorbidity index >4 (adjusted hazard ratio, 3.80; 95% confidence interval, 1.52–9.47) was associated with 30-day mortality, whereas ID consultation within 48 hours (0.22, 0.10–0.48) and performance of source control interventions within 48 hours (0.12, 0.03–0.50) were associated with improved outcome. Complicated disease was not associated with 30-day mortality (0.39, 0.10–1.46).

**Conclusions:**

The proposed definition for complicated CoNS bacteremia failed to identify patients at higher risk for mortality in our cohort. Our findings highlight the importance of ID consultation in guiding antimicrobial treatment and recommending source control interventions for patients with CoNS bacteremia.

## Teaser

Based on the proposed definition, 195 of 326 episodes (60%) had complicated coagulase-negative staphylococcal bacteremia. The overall 30-day mortality was 9% (29 episodes). No difference on 30-day mortality was observed among complicated and uncomplicated disease (10% vs 7%; *P* = .327).

Coagulase-negative staphylococci (CoNS) are a frequent cause of bacteremia, particularly in hospitalized patients with catheters and indwelling devices [1]. However, because they are common skin commensals, CoNS are often isolated from blood cultures, with most cases representing contamination rather than true infection [2–5].

Because of CoNS’ low virulence and association with catheter-related infections, the bacteremia's 30-day mortality is lower than that of *Staphylococcus aureus* bacteremia [1]. However, studies investigating predictors of mortality in CoNS bacteremia are scarce. Factors previously associated with increased mortality include advanced age, severity of infection, comorbidities, inappropriate antimicrobial therapy, and lack of source control interventions [3–7].

Most CoNS infections are catheter-related, and guidelines recommend catheter removal as an essential source control measure. In cases where the catheter is removed and there is no evidence of metastatic infection, septic thrombophlebitis, or infective endocarditis, a short course of antimicrobial treatment (3–5 days) is sufficient [8]. Some studies even suggest that catheter removal alone, without additional antimicrobial therapy, may be a safe approach [9, 10], underscoring the importance of this intervention. For patients with retained long-term catheters, a prolonged course of antimicrobial treatment (10–14 days) combined with antibiotic lock therapy is recommended to achieve effective source control through high-concentration local antibiotic administration [8].

Unlike *S. aureus* bacteremia, there is no widely accepted definition of complicated disease for bacteremia caused by other species, including CoNS [11]. Recently, Varisco et al proposed a definition of complicated CoNS bacteremia, modeled on the Infectious Diseases Society of America's criteria for complicated *S. aureus* bacteremia [11]. The proposed definition includes 6 criteria: persistent fever or persistent bacteremia lasting ≥72 hours after the initiation of antimicrobial therapy, presence of implanted devices, metastatic complications, infective endocarditis, and, in catheter-related infections, failure to remove the catheter within 48 hours of bacteremia onset [6]. Varisco et al found that approximately half of the patients met the criteria for complicated CoNS bacteremia and that these patients had higher mortality compared to those with uncomplicated bacteremia (22% vs 12%, *P* = .004) [6].

This study aims to identify predictors of mortality in patients with CoNS bacteremia and evaluate the proposed definition of CoNS complicated bacteremia.

## MATERIALS AND METHODS

This retrospective study was conducted at Lausanne University Hospital, Switzerland, from 2014 to 2023. The study included nonduplicate cases from 2 cohorts: the bacteremia cohort (2015–2021; retrospective inclusion) and the cohort of patients with suspected infective endocarditis (2014–2023; retrospective inclusion: 2015–2017; prospective inclusion: 2018–2023). The study was approved by the ethics committee of the Canton of Vaud (CER-VD 2021-02516, CER-VD 2017-02137).

Inclusion criteria were adult patients (≥18 years old), presence of true CoNS bacteremia and absence of patient's decline of the use of their data. Exclusion criterion consisted of cases with incomplete medical records (including patients transferred to other hospitals at the onset of infection without follow-up data).

Blood cultures were incubated using the BacT/ALERT System (bioMerieux, Marcy l'Etoile, France). Species identification was performed using matrix-assisted laser desorption-ionization time of flight mass spectrometry (Bruker Daltonics, Bremen, Germany) from 7:00 Am to 7:00 Pm. Susceptibility results were obtained from the microbiology laboratory database and assessed in accordance with the European Committee on Antimicrobial Susceptibility Testing criteria [12].

The primary outcome of the study was the 30-day crude mortality. Data on demographics (age, sex), comorbidities, Charlson Comorbidity Index, antimicrobial treatment, source control, presence of sepsis or septic shock, and the site of infection were retrieved from patients’ electronic health records by an infectious diseases consultant (M. P. O.).

At our institution, infectious diseases (ID) consultants are notified of patients with positive blood cultures after species identification. Unlike *S. aureus* and *Candida* spp., ID consultation is not mandatory for bacteremia caused by other microorganisms [13, 14]. Follow-up blood cultures to confirm bacteremia clearance were performed in patients with persistent symptoms or suspected of infective endocarditis.

Primary outcome was overall 30-day mortality. Secondary outcomes were 120-day mortality and 120-day recurrence of bacteremia from the same staphylococcal species. The date of collection of the first positive blood culture was defined as the onset of bacteremia. A new episode was included if more than 120 days had elapsed since the initial bacteremia. Bacteremia cases were classified as community-acquired, healthcare-associated, or nosocomial, following the criteria established by Friedman et al [15]. Sepsis and septic shock were defined according to the Sepsis-3 International Consensus definition [16]. The diagnosis of infective endocarditis was established by the endocarditis team. The site of infection was determined by the ID consultant based on clinical, radiological, microbiological, and operative findings. Appropriate antimicrobial treatment was defined as the initiation of at least 1 antimicrobial agent with in vitro activity against the causative pathogen. Source control deemed necessary in the following scenarios: (1) removal of venous catheter in cases with catheter-related bacteremia or bacteremia of unknown origin with a venous catheter in place; (2) antibiotic lock therapy for long-term central venous catheters that were not removed [8]; or (3) imaging-guided or surgical drainage of infected collections or material. Complicated disease was defined based on the most recent proposed criteria [6].

SPSS version 26.0 (SPSS, Chicago, IL, USA) was used for data analyses. Categorical variables were analyzed using the chi-squared or Fisher exact test and continuous variables with Mann–Whitney *U* test. Univariate logistic regression models were assessed with 30-day mortality as the dependent variable. Clinically relevant noncollinear covariates, assessed through variance inflation factor, were used in multivariable analysis. After checking Cox assumptions, a multivariable Cox proportional hazards regression models were performed with 30-day mortality as the time-to-event. Hazard ratios (HRs) and 95% confidence intervals (CIs) were calculated to evaluate the strength of any association. All statistic tests were 2-tailed and *P* < .05 was considered statistically significant. Survival curves were generated to compare complicated and uncomplicated bacteremia, as well as episodes with and without appropriate source control within 48 hours of bacteremia onset.

## RESULTS

**Figure 1.**
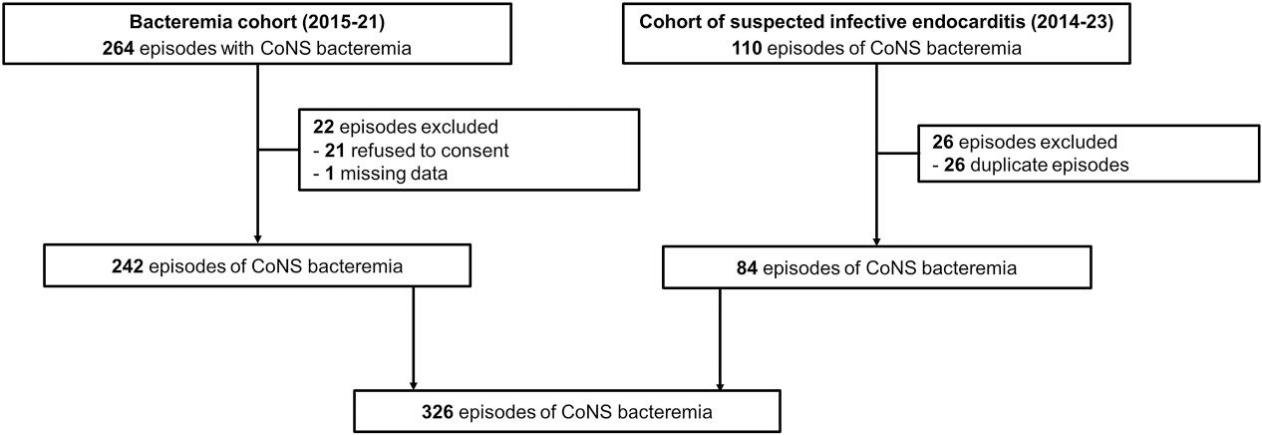
Flowchart of patients’ selection. Abbreviation: CoNS, coagulase-negative staphylococci.

A total of 374 episodes of CoNS bacteremia were identified across both cohorts, with 326 nonduplicate episodes included involving 296 patients (Figure 1); the 30 subsequent episodes of bacteremia by the same staphylococcal species occurred at a median of 6 months from the previous episode (range, 5–53 months) (Supplementary Table 1). *Staphylococcus epidermidis* (250 episodes; 77%) predominated, followed by *Staphylococcus lugdunensis* (33; 10%). A total of 223 (68%) isolates demonstrated methicillin resistance.

From 326 eligible episodes, most infections were catheter-related bacteremia (233; 68%), followed by infective endocarditis (50; 15%). Sepsis occurred in 102 (31%) episodes. Follow-up blood cultures until sterilization were performed in 319 (98%) episodes; among them, 73 (23%) had persistent bacteremia for at least 48 hours.

ID consultation was provided in 307 (94%) episodes; it was delivered within 48 hours from bacteremia onset in 285 (87%) of these episodes. Antimicrobial treatment was initiated before the results of the blood cultures in 207 (64%) episodes, with 286/326 (88%) episodes receiving appropriate antimicrobial treatment within 48 hours. Source control was deemed necessary in 275 (84%) episodes and was performed in 261/275 (95%) during the stay; in 167/275 (61%) episodes such interventions were performed. within 48 hours of bacteremia onset.

Based on the proposed definition, 195 (60%) episodes had complicated disease. Among the 242 episodes from the bacteremia cohort, 127 (52%) had complicated disease. Episodes with complicated bacteremia had higher Charlson Comorbidity Index compared to those with uncomplicated disease (median of 5; interquartile range [IQR]: 2–6) *versus* 4 [IQR: 3–7] points; *P* = .002) (Table 1). Half of the catheter-related bacteremias (116 of 233) were classified as complicated.

**Table 1.** Comparison of Episodes With Complicated and Uncomplicated Disease

	Uncomplicated (n = 131)	Complicated (n = 195)	*P*
Demographics			
Male	93 (71)	131 (67)	.543
Age (y)	62 (48–72)	66 (55–77)	.002
Age >60 y	68 (52)	126 (65)	.029
Comorbidities			
Malignancy (solid organ or hematologic)	72 (55)	74 (38)	.003
Immunosuppression^a^	50 (38)	70 (36)	.726
Diabetes mellitus	20 (15)	49 (25)	.038
Chronic kidney disease (moderate or severe)	18 (14)	53 (27)	.004
Obesity (body mass index ≥30 kg/m^2^)	21 (16)	42 (22)	.253
Chronic obstructive pulmonary disease	18 (14)	29 (15)	.873
Congestive heart failure	7 (5)	25 (13)	.035
Cirrhosis	9 (7)	17 (9)	.678
Charlson Comorbidity Index	4 (2–6)	5 (3–7)	.002
Charlson Comorbidity Index >4	59 (45)	107 (55)	.091
Setting of bacteremia onset			
Community	12 (9)	54 (28)	
Healthcare-associated	11 (8)	34 (17)	
Nosocomial	108 (82)	107 (55)	<.001^b^
Microbiological data			
Methicillin-resistant	90 (69)	133 (69)	1.000
Types of coagulase negative staphylococci			
*Staphylococcus epidermidis*	104 (79)	146 (75)	.344^c^
*Staphylococcus lugdunensis*	8 (6)	25 (13)	
Other	20 (15)	27 (14)	
Polymicrobial bacteremia	23 (18)	19 (10)	.044
Manifestations	38 (13)	4 (14)	.777
Fever	112 (86)	154 (80)	.188
Sepsis	38 (29)	64 (33)	.543
Septic shock	4 (3)	8 (4)	.768
Type of infection			
Catheter-related	117 (89)	116 (60)	<.001
Bone and joint infection	0 (0)	19 (10)	<.001
Endocarditis	0 (0)	50 (24)	<.001
Vascular graft infection	0 (0)	7 (7)	.044
Other foci	14 (12)	29 (28)	.318
Management			
Infectious diseases consultation	118 (90)	189 (97)	.014
Infectious diseases consultation within 48 h	106 (81)	179 (92)	.006
Source control			
Not warranted	7 (5)	44 (23)	
Warranted and performed within 48 h	119 (91)	48 (25)	
Warranted, but not performed within 48 h	5 (4)	103 (53)	<.001^d^
Antimicrobial initiation before result of blood cultures	85 (64)	122 (63)	.907
Antimicrobial initiation within 48 h	127 (97)	179 (92)	.063
Appropriate antimicrobial within 48 h	120 (92)	166 (85)	.087
Outcome			
30-d mortality	9 (7)	20 (10)	.327
120-d mortality	24 (18)	42 (22)	.487
Recurrence of bacteremia within 120 d	3 (2)	9 (5)	.374

Data are depicted as number (percentage) or median (Q1–3).

^a^Ongoing immunosuppressive treatment at bacteremia onset, intravenous chemotherapy in the 30 d before bacteremia onset, AIDS, neutropenia and asplenia.

^b^Comparison of nosocomial bacteremia with both community- and healthcare-associated.

^c^Comparison of episodes of *S. epidermidis* bacteremia with those caused by other staphylococcal species.

^d^Comparison among episodes warranting source control interventions between those that had such interventions performed within 48 h and those that did not.

**Figure 2.**
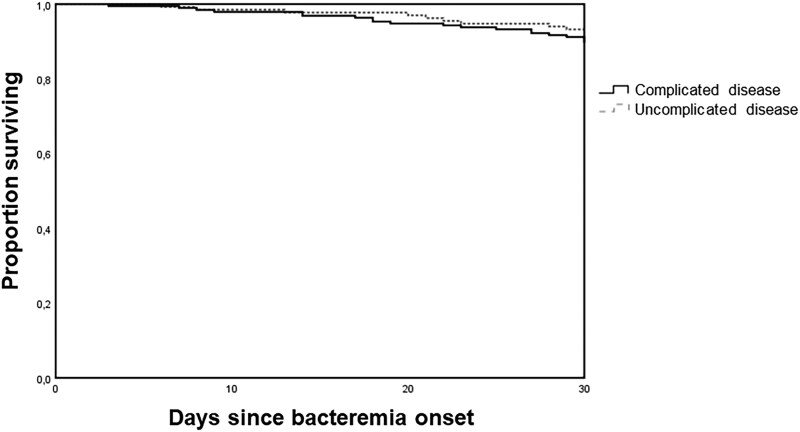
Kaplan–Meier curves of the survival probability of patients with complicated and uncomplicated bacteremia by coagulase-negative staphylococci.

The overall 30-day mortality was 9% (29 episodes). Mortality and recurrence of bacteremia from the same staphylococcal species at 120 days was observed in 66 (20%) and 12 (4%) episodes. No difference was observed among episodes with complicated and uncomplicated disease on 30-day mortality (10% vs 7%; *P* = .327), 120-day mortality (22% vs 18%; *P* = .487), or 120-day recurrence of bacteremia (5% vs 2%; *P* = .374). Figure 2 shows 30-day survival probability curves of patients with complicated and noncomplicated bacteremia (log-rank test *P* = .271).

The comparison of 30-day survivors and nonsurvivors is shown in Table 2. Episodes due to *S. lugdunensis* had a 30-day mortality of 15% (5/33), compared to 8% (24/293) for other species (*P* = .194). The results from the Cox multivariable regression model (Table 3) showed that a Charlson Comorbidity Index >4 (*P* = .004; adjusted HR [aHR], 3.80; 95% CI, 1.52–9.47) was associated with 30-day mortality, whereas ID consultation within 48 hours (*P* < .001; aHR, 0.22; 95% CI, .10–.48), and performance of source control interventions within 48 hours (*P* = .003; aHR, 0.12; 95% CI, .03–.50) were associated with improved outcome. Complicated disease (*P* = .161; aHR, 0.39; 95% CI, .10–1.46) was not associated with 30-day mortality.

**Table 2.** Comparison of Survivors and Not Survivors at Day 30

	Survivors (n = 297)	Nonsurvivors (n = 29)	*P*
Demographics			
Male	209 (70)	15 (52)	.057
Age (y)	64 (52–75)	70 (56–80)	.066
Age >60 y	173 (58)	21 (72)	.167
Comorbidities			
Malignancy (solid organ or hematologic)	138 (47)	8 (28)	.077
Immunosuppression^a^	112 (38)	8 (28)	.319
Diabetes mellitus	58 (20)	11 (38)	.030
Chronic kidney disease (moderate or severe)	59 (20)	12 (41)	.016
Obesity (body mass index ≥30 kg/m^2^)	57 (19)	6 (21)	.808
Chronic obstructive pulmonary disease	43 (15)	4 (14)	1.000
Congestive heart failure	29 (10)	3 (10)	1.000
Cirrhosis	21 (7)	5 (17)	.068
Charlson Comorbidity Index	4 (3–7)	6 (5–9)	.001
Charlson Comorbidity Index >4	143 (48)	23 (79)	.002
Setting of bacteremia onset			
Community	58 (20)	8 (28)	
Healthcare-associated	43 (15)	2 (7)	
Nosocomial	196 (66)	19 (66)	1.000^b^
Vascular access	245 (83)	23 (79)	0.617
Central venous catheters	204 (69)	19 (66)	.834
Peripheral venous catheters	54 (18)	6 (21)	.801
Implanted devices^c^	91 (31)	10 (35)	.677
Prosthetic valve	30 (10)	3 (10)	1.000
Cardiac implantable electronic device	25 (8)	2 (7)	1.000
Prosthetic joint	30 (10)	3 (10)	1.000
Osteosynthesis/spondylodesis	15 (5)	0 (0)	.378
Vascular graft	26 (9)	7 (24)	.018
Microbiological data			
Methicillin-resistant	202 (68)	21 (72)	.682
Types of coagulase negative staphylococci			
*Staphylococcus epidermidis*	229 (77)	21 (72)	.645^d^
*Staphylococcus lugdunensis*	28 (9)	5 (17)	
Other	44 (15)	3 (10)	
Persistent bacteremia (≥48 h)	64 (22)	9 (31)	.248
Persistent bacteremia (≥72 h)^c^	38 (13)	6 (21)	.253
Polymicrobial bacteremia	38 (13)	4 (14)	.777
Manifestations	38 (13)	4 (14)	.777
Fever	245 (83)	22 (76)	.446
Persistent fever ≥72 h^c^	15 (5)	3 (10)	.208
Sepsis	89 (30)	13 (45)	.140
Septic shock	11 (4)	1 (3)	1.000
Embolic events	20 (7)	6 (21)	.019
Type of infection			
Catheter-related	217 (73)	16 (55)	.052
Bone and joint infection	17 (6)	2 (7)	.681
Septic arthritis	3 (1)	0 (0)	1.000
Osteomyelitis vertebral or nonvertebral	8 (3)	0 (0)	1.000
Prosthetic joint infection	4 (1)	0 (0)	1.000
Osteosynthesis/spondylodesis infection	3 (1)	0 (0)	1.000
Endocarditis^c^	43 (15)	7 (24)	.178
Native valve	22 (7)	5 (17)	.078
Prosthetic valve	17 (6)	3 (10)	.404
Cardiac implantable electronic device lead	8 (3)	1 (3)	.572
Vascular graft infection	5 (2)	2 (7)	.121
Other foci	35 (12)	8 (28)	.038
Related to prosthetic material	253 (85)	22 (76)	.186
Metastatic complications^c^	40 (14)	10 (35)	.006
Complicated disease	175 (59)	20 (69)	.327
Management			
Infectious diseases consultation	282 (95)	25 (86)	.076
Infectious diseases consultation within 48 h	267 (90)	18 (62)	<.001
Source control			
Not warranted	47 (16)	4 (14)	
Warranted and performed within 48 h	159 (54)	8 (28)	
Warranted, but not performed within 48 h	91 (31)	17 (59)	.003^e^
Absence of catheter removal within 48 h	68 (23)	10 (35)	.174
Antimicrobial initiation before result of blood cultures	188 (63)	19 (66)	1.000
Antimicrobial initiation within 48 h	273 (92)	28 (97)	.494
Appropriate antimicrobial within 48 h	263 (89)	23 (79)	.146

Data are depicted as number (percentage) or median (Q1–3).

^a^Ongoing immunosuppressive treatment at bacteremia onset, intravenous chemotherapy in the 30 d prior to bacteremia onset, AIDS, neutropenia and asplenia.

^b^Comparison of nosocomial bacteremia with both community and healthcare-associated.

^c^Criteria for complicated bacteremia.

^d^Comparison of episodes of *S. epidermidis* bacteremia with those caused by other staphylococcal species.

^e^Comparison among episodes warranting source control interventions between those that had such interventions performed within 48 h and those that did not.

**Table 3.** Univariable and Multivariable Cox Proportional Hazard Regression of 30-Day Mortality Among Patients With Bacteremia Due to Coagulase Negative Staphylococci

	Univariable Analysis		Multivariable Cox Regression	
	*P*	HR (95% CI)	*P*	aHR (95% CI)
Charlson Comorbidity Index >4	.002	4.04 (1.64–9.91)	.004	3.80 (1.52–9.47)
Infectious diseases consultation within 48 h	<.001	.21 (.10–.45)	<.001	.22 (.10–.48)
Complicated disease	.327	1.59 (.70–3.62)	.161	.39 (.10–1.46)
Source control				
Warranted, but not performed within 48 h	Reference		Reference	
Warranted and performed within 48 h	.004	.29 (.13–.67)	.003	.12 (.03–.50)
Not warranted	.197	.49 (.16–1.45)	.168	.45 (.14–1.41)

Abbreviations: aHR, adjusted hazard ratio; CI, confidence interval; HR, hazard ratio.

**Figure 3.**
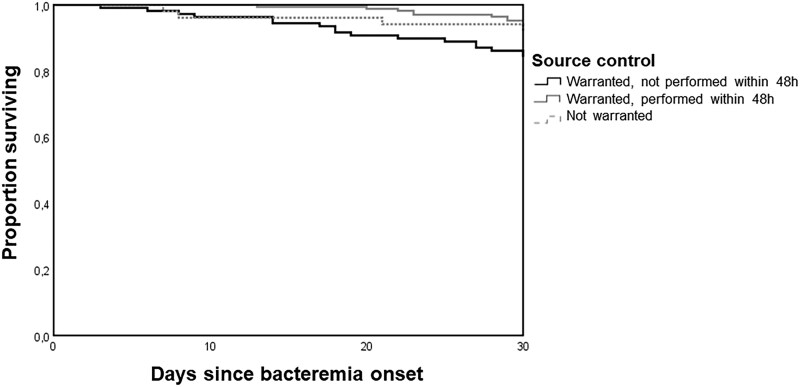
Kaplan–Meier curves of the survival probability of patients with bacteremia by coagulase-negative staphylococci according to need and performance of early source control.

Figure 3 shows survival probability curves for episodes with CoNS bacteremia based on the need for and performance of early source control. Early source control was linked to a more favorable outcome when compared to episodes where it was warranted but not performed (log-rank test *P* = .002).

Among the 101 (31%) episodes with implantable devices, 40 (40%) had an infection of the device. In the remaining 61 episodes (60%), device infection was excluded, and these cases were treated for less than 14 days; 2 patients died while receiving antimicrobial therapy. Of the remaining 59 episodes, only 2 patients experienced a recurrence of CoNS bacteremia within 120 days, both of which were attributed to new catheter-related bacteremia.

## DISCUSSION

In this study, the proposed definition for complicated CoNS bacteremia failed to identify patients at higher risk of adverse outcomes.

The 30-day mortality rate was 9%, which is lower than the rates reported in previous studies (11%–24%), and lower to that of *S. aureus* bacteremia from our institution (15%) [1, 3–7, 13, 17]. The most plausible explanation for the lower mortality observed in our study is the high rate of ID consultations, which led to improved outcomes by optimizing antimicrobial therapy and source control measures. Notably, ID consultations were provided in 94% of cases, with 87% occurring within 48 hours. In contrast, a previous study reported ID consultation in only 68% of cases during hospitalization [17]. In the current study, 88% of patients received appropriate antimicrobial therapy within 48 hours, a rate comparable to those reported in earlier studies within a 72-hour timeframe (84%–88%) [3, 5]. Additionally, source control procedures were performed more frequently in our cohort than in prior studies [4, 6]. To our knowledge, this is the first study to assess and demonstrate the beneficial role of early ID consultation in managing CoNS bacteremia. These findings support adding CoNS bacteremia to the growing list of pathogens (*S. aureus*, enterococci, streptococci, *Pseudomonas aeruginosa*, *Candida* spp.) for which ID consultation is strongly recommended [13, 18–21].

In addition to early ID consultation, prompt source control procedures were associated with improved outcomes. The absence of indicated source control interventions has previously been linked to worse outcomes in patients with CoNS bacteremia [4, 6] and bacteremia caused by other pathogens [13, 20, 21]. This aspect of management is particularly critical for CoNS bacteremia, as most infections (84%) are associated with prosthetic material. Effective management may require device removal, debridement, or antibiotic lock therapy in cases of catheter-related bacteremia with retained long-term catheters [8]. In patients with uncomplicated catheter-related bacteremia, catheter removal alone, without concomitant antimicrobial therapy, may be sufficient to control the infection [9, 10].

As expected, a high Charlson Comorbidity Index was associated with worse outcomes. Previous studies on CoNS bacteremia have also identified advanced age and specific comorbidities, such as chronic renal or liver disease, cerebrovascular disease, and immunosuppression, as predictors of mortality [3, 6]. However, in contrast to earlier findings, the severity of infection was not significantly associated with adverse outcomes in our cohort [4, 6, 7]. This discrepancy is likely attributable to the low prevalence of septic shock in our study (4%) compared to prior studies (7%–20%) [6, 7].

Based on the recent proposed definition for complicated CoNS bacteremia, in our study, a similar percentage (60%) of patients had complicated disease compared to that of Varisco et al (54%) [6]. However, in contrast to their findings, the presence of complicated disease in our cohort was not associated with worse outcomes, including 30-day, 120-day mortality, or 120-day bacteremia recurrence. Several factors may explain this discrepancy. First, 3 of the criteria for complicated bacteremia—absence of early catheter removal, persistent fever, and persistent bacteremia—reflect a common underlying issue: the lack of source control, and to a lesser extent, inappropriate antimicrobial therapy [6]. Absence of prompt source control interventions was associated with higher mortality both in our study and in previous literature [4, 6]. However, in our cohort, neither persistent bacteremia nor persistent fever alone were linked to worse outcomes. In Varisco et al, the impact of persistent bacteremia may have been overestimated because follow-up blood cultures were obtained in only 27% of patients, with 36% of those showing persistent bacteremia lasting at least 72 hours [6]. In contrast, follow-up blood cultures were almost universally performed in our study (98%), with only 23% of patients exhibiting persistent bacteremia within the same timeframe.

Moreover, the presence of implanted devices in the absence of clinical or radiological signs of infection should not automatically be considered a criterion for complicated disease. In our cohort, for patients where device infection was excluded and treated with a short course of antimicrobial therapy (<14 days), none experienced device-related infections within the 120 days.

*S. epidermidis* was the predominant species, limiting the ability to fully assess the impact on mortality of other species, including *S. lugdunensis*. As expected, *S. lugdunensis* was more prevalent among episodes with complicated diseases and showed a higher 30-day mortality compared to other species but this difference was not statistically significant. This finding is consistent with the study by Yukawa et al [22], which also reported a numerically higher 30-day mortality for *S. lugdunensis* bacteremia (15%) compared to *S. epidermidis* (8%), though the difference was not statistically significant because of the small sample size. The increased rates of complicated disease and mortality observed with *S. lugdunensis* may be due to its greater number of virulence factors that enhance its pathogenicity compared to other CoNS species. However, *S. lugdunensis* lacks key virulence factors characteristic of *S. aureus*, such as coagulase [23].

This study has several limitations. First, it is a retrospective, single-center study conducted at a tertiary hospital, which tends to manage more complex cases; furthermore, we used patients from the cohort of suspected IE, potentially leading to an overrepresentation of cases at high risk for IE. Consequently, our findings may not fully represent the epidemiology of CoNS bacteremia in nontertiary care settings. Second, numerous confounding factors can influence decisions regarding early source control interventions, including care-limiting decisions or withdrawal of care, potentially introducing survivorship bias. However, no patient died within 48 hours from bacteremia onset, and only 2 were considered for withdrawal of care within the same timeframe.

Our study highlights the importance of a comprehensive approach to managing CoNS bacteremia, which includes early interventions such as ID consultation, tailored antimicrobial therapy, and timely source control measures when indicated. The proposed definition for complicated CoNS bacteremia failed to identify patients at higher risk for mortality in our cohort. Further studies are needed to refine the definition and improve its ability to identify patients with an elevated risk of poor outcomes.

## Supplementary Material

ciaf119_Supplementary_Data

## Supplementary Data

Supplementary materials are available at *Clinical Infectious Diseases* online. Consisting of data provided by the authors to benefit the reader, the posted materials are not copyedited and are the sole responsibility of the authors, so questions or comments should be addressed to the corresponding author.

## Note

***Author Contributions*.** M. P. O. conceived the idea and collected the patients’ data. B. G. supervised the project. M. P. O. performed the analysis and interpreted the results and wrote the manuscript. All authors contributed to manuscript revision and read and approved the submitted version.
