# Supplementary material for: Validation of the Proposed Definition for Complicated Coagulase-negative Staphylococcal Bacteremia
Source: Clin Infect Dis. 2025 Mar 13;80(6):1208–15. doi: 10.1093/cid/ciaf119 (PMC12272850; doi:10.1093/cid/ciaf119)
Supplement: ciaf119_Supplementary_Data [file ciaf119_supplementary_data.pdf]

Supplementary Table 1. Diagnoses of initial and subsequent episodes of bacteremia caused by the same staphylococcal species, occurring at least 120 days apart.

	**Species**	**Diagnosis of the initial episode**	**Diagnosis of the subsequent episode**	**Months elapsing between episodes**
1	*S. epidermidis*	Catheter-related[a]	Catheter-related	5
2	*S. epidermidis*	Catheter-related[a]	Catheter-related	5
3	*S. epidermidis*	Catheter-related[a]	Catheter-related	5
4	*S. epidermidis*	Catheter-related[a]	Catheter-related	5
5	*S. epidermidis*	Catheter-related[a]	Catheter-related	5
6	*S. epidermidis*	Catheter-related[a]	Catheter-related	5
7	*S. epidermidis*	Catheter-related[a]	Catheter-related	5
8	*S. epidermidis*	Catheter-related[a]	Catheter-related	6
9	*S. epidermidis*	Catheter-related[a]	Catheter-related	6
10	*S. epidermidis*	Catheter-related[a]	Catheter-related	6
11	*S. epidermidis*	Catheter-related[a]	Catheter-related	7
12	*S. epidermidis*	Catheter-related[a]	Catheter-related	8
13	*S. epidermidis*	Catheter-related[a]	Catheter-related	8
14	*S. epidermidis*	Catheter-related[a]	Catheter-related	9
15	*S. epidermidis*	Catheter-related[a]	Catheter-related	11
16	*S. epidermidis*	Catheter-related[a]	Catheter-related	13
17	*S. epidermidis*	Catheter-related[a]	Catheter-related	14
18	*S. epidermidis*	Catheter-related	Catheter-related	19
19	*S. epidermidis*	Catheter-related[a]	Catheter-related	21
20	*S. epidermidis*	Endocarditis	Catheter-related	7
21	*S. epidermidis*	Endocarditis	Catheter-related	16
22	*S. epidermidis*	Prosthetic-joint infection	Endocarditis	6
23	*S. epidermidis*	Prosthetic-joint infection	Catheter-related	8
24	*S. epidermidis*	Catheter-related[a]	Vascular graft infection	53
25	*S. epidermidis*	Catheter-related[a]	Endocarditis	19
26	*S. lugdunensis*	Catheter-related[a]	Catheter-related	5
27	*S. lugdunensis*	Catheter-related[a]	Catheter-related	5
28	*S. lugdunensis*	Catheter-related[a]	Catheter-related	6
29	*S. haemolyticus*	Catheter-related[a]	Catheter-related	5
30	*S. hominis*	Catheter-related[a]	Vascular graft infection	6

[a]implicated catheter was removed during antimicrobial treatment of the initial episode

For the 23 patients in whom both the initial and subsequent infections were catheter-related, the latter was considered a new infection since, in all but one case, the implicated catheter from the initial episode had been removed. In the single patient whose catheter was not removed during the initial episode, the subsequent episode of catheter-related bacteremia occurred 19 months later.
